# Evolution equation for quantum coherence

**DOI:** 10.1038/srep29260

**Published:** 2016-07-07

**Authors:** Ming-Liang Hu, Heng Fan

**Affiliations:** 1 School of Science, Xi’an University of Posts and Telecommunications, Xi’an 710121, China; 2 Beijing National Laboratory for Condensed Matter Physics, Institute of Physics, Chinese Academy of Sciences, Beijing 100190, China; 3 Collaborative Innovation Center of Quantum Matter, Beijing 100190, China

## Abstract

The estimation of the decoherence process of an open quantum system is of both theoretical significance and experimental appealing. Practically, the decoherence can be easily estimated if the coherence evolution satisfies some simple relations. We introduce a framework for studying evolution equation of coherence. Based on this framework, we prove a simple factorization relation (FR) for the *l*_1_ norm of coherence, and identified the sets of quantum channels for which this FR holds. By using this FR, we further determine condition on the transformation matrix of the quantum channel which can support permanently freezing of the *l*_1_ norm of coherence. We finally reveal the universality of this FR by showing that it holds for many other related coherence and quantum correlation measures.

Quantum coherence, an embodiment of the superposition principle of states, lies at the heart of quantum mechanics, and is also a major concern of quantum optics^1^. Physically, coherence constitutes the essence of quantum correlations (e.g., entanglement^2^ and quantum discord^3^) in bipartite and multipartite systems which are indispensable resources for quantum communication and computation tasks. It also finds support in the promising subject of thermodynamics^4^,^5^,^6^,^7^,^8^ and quantum biology^9^.

Clarifying the decoherence mechanism of an noisy system is an important research direction of quantum mechanics. But due to the lack of rigorous coherence measures, studies in this subject were usually limited to the qualitative analysis. Sometimes, coherence behaviors were also analyzed indirectly via various quantum correlation measures^3^. However, coherence and quantum correlations are in fact different. Very recently, the characterization and quantification of quantum coherence from a mathematically rigorous and physically meaningful perspective has been achieved^10^. This sets the stage for quantitative analysis of coherence, which were carried out mainly around the identification of various coherence monotones^11^,^12^,^13^,^14^,^15^,^16^ and their calculation^17^. Some other progresses about coherence quantifiers include their connections with quantum correlations^18^,^19^,^20^, their behaviors in noisy environments^21^,^22^, their local and nonlocal creativity^23^,^24^, their distillation^25^,^26^ and the role they played in the fundamental issue of quantum mechanics^27^,^28^,^29^,^30^.

One major goal of quantum theory is to find effective ways of maintaining the amount of coherence within a system. The reason is twofold. First, coherence represents a basic feature of quantum states, and underpins all forms of quantum correlations^1^. Second, coherence itself is a precious resource for many new quantum technologies, but the unavoidable interaction of quantum devices with the environment often decoheres the input states and induces coherence loss, hence damage the superiority of these quantum technologies^31^.

Looking for general law determining the evolution equation of coherence can facilitate the design of effective coherence preservation schemes. Remarkably, the evolution equations for certain entanglement monotones (or their bounds)^32^,^33^,^34^,^35^,^36^,^37^,^38^,^39^,^40^ and geometric discords^41^ were found to obey the factorization relation (FR) for specific initial states. Then, it is natural to ask whether there exists similar FR for various coherence monotones. In this work, we aimed at solving this problem. We first classify the general *d*-dimensional states into different families, and then prove a FR which holds for them. By employing this FR, we further identified condition on the quantum channel for freezing coherence. We also showed that this FR applies to many other coherence and correlation measures. These results are hoped to add another facet to the already rich theory of decoherence, and shed light on revealing the interplay between structures of quantum channel and geometry of the state space, as well as how they determine quantum correlation behaviors of an open system.

## Results

### Coherence measures

By establishing rigorously the sets 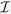 of incoherent states which are diagonal in the reference basis {|*i*〉}_*i*=1,…,*d*_, and incoherent operations Λ specified by the Kraus operators {*E*_*l*_} which map 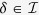 into 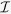, Baumgratz *et al*.^10^ presented the defining properties for an information-theoretic coherence measure *C*: (1) *C*(*ρ*) ≥ 0 for all states *ρ*, and *C*(*δ*) = 0 iff 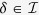. (2) Monotonicity under the actions of Λ, *C*(*ρ*) ≥ *C*(Λ(*ρ*)). (3) Monotonicity under selective incoherent operations on average, i.e., 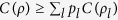, where 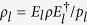, and 
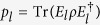
 is the probability of obtaining the outcome *l*. (4) Convexity, 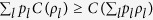, with *p*_*l*_ ≥ 0 and 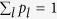.

There are several coherence measures satisfying the above conditions. They are the *l*_1_ norm and relative entropy^10^, the Uhlmann fidelity^12^, the intrinsic randomness^14^, and the robustness of coherence^42^. In this work, we concentrate mainly on the *l*_1_ norm of coherence, which is given by 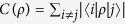 in the basis {|*i*〉}_*i*=1,…,*d*_^10^, and will mention other coherence measures if necessary.

### FR for quantum coherence

Consider a general *d*-dimensional state in the Hilbert space 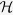, with the density matrix


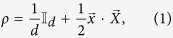


where 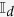 is the *d* × *d* identity matrix, 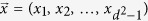, 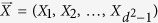, *x*_*i*_ = Tr(*ρX*_*i*_), and *X*_*i*_ ∝ *T*_*i*_. Here, {*T*_*i*_} are generators of the Lie algebra SU(*d*). They can be represented by the *d* × *d* traceless Hermitian matrices which satisfy 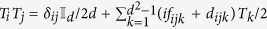, with *f*_*ijk*_ (*d*_*ijk*_) being the structure constants that are completely antisymmetric (symmetric) in all indices^43^,^44^. If one arranges 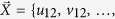

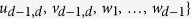
, then


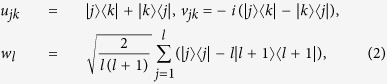


where *j*, *k* ∈ {1, 2, …, *d*} with *j* < *k*, and *l* ∈ {1, 2, …, *d* − 1}. Clearly, {*X*_*i*_} satisfy 
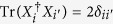
. Moreover, the notation *i* appeared in *v*_*jk*_ is the imaginary unit.

For *ρ* represented as Eq. (1), 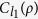 can be derived as


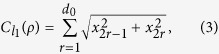


where *d*_0_ = (*d*^2^ − *d*)/2, and *x*_*l*_ related to *w*_*l*_ which is diagonal in the basis {|*i*〉}_*i*=1,…,*d*_ do not contribute to 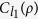.

To investigate evolution equation of coherence, we suppose the system *S* of interest interacts with its environment *E*, then by considering *S* and *E* as a whole for which their evolution is unitary, the reduced density matrix for *S* is obtained by tracing out the environmental degrees of freedom, 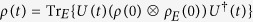. In terms of the master equation description, the equation of motion of *ρ* can be written in a local-in-time form^31^


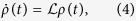


with 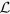 being the Louville super-operator which may be time independent or time dependent.

As it has been shown that for any master equation which is local in time, whether Markovian, non-Markovian, of Lindblad form or not, one can always construct a linear map which gives 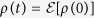 (the opposite case may not always be true), and the linear map can be expressed in the Kraus-type representations^45^. If the map 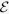 is completely positive and trace preserving (CPTP), then one can explicitly construct the Kraus operators {*E*_*μ*_} such that


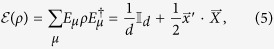


where elements of 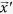 for 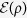 are given by 
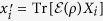
.

For convenience of later discussion, we turn to the Heisenberg picture to describe 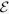 via the map 
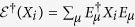
, which gives 
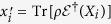
. As an Hermitian operator 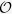 on 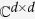 can always be decomposed as 
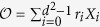
 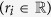, 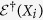 can be further characterized by the transformation matrix *T* defined via


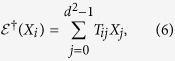


where 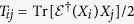, and here we denote by 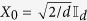. Clearly, *T*_00_ = 1, and *T*_0*j*_ = 0 for *j* ≥ 1. This further gives 
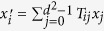
.

**Figure 1:**
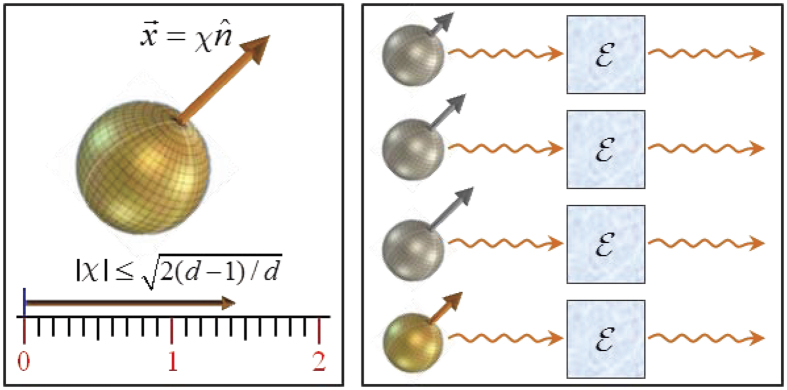
States of the same family 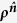 are represented by the characteristic vectors 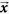 along the same or opposite directions (left). When 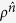 traverse a quantum channel 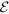 (right), their decoherence process can be described qualitatively by that of 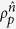 with the unit vector 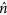 (the bottommost golden one).

To present our central result, we first classify the states *ρ* into different families: 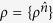, with


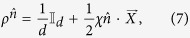


and 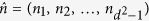 is a unit vector in 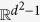, while *χ* is a parameter satisfying 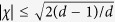 as 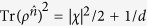. By this classification scheme, different families of states are labeled by different unit vectors 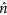, while states belong to the same family are characterized by a common 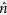, and can be distinguished by different multiplicative factors *χ* (see Fig. 1). That is to say, 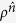 represents states with the characteristic vectors 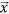 along the same or completely opposite directions but possessing different lengths.

While 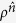 is fully described by 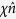, and the action of 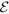 on it can be written equivalently as the map: 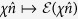, a measure *Q* may only be function of 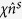, i.e., 
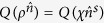
, with 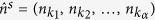 (*α* ≤ *d*^2^ − 1). Then as one can always make *Q*_max_ ≥ 1 (otherwise, one can normalize it by multiplying a constant to it), we have the following lemma.

**Lemma 1.** *For any quantum measure of* 
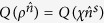
 *that can be factorized as* 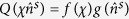, *and quantum channel* 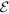 *that gives the map* 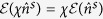, *the FR*


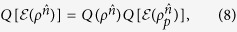


*holds*, *where f*(*χ*) *and* 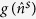 *are functionals of χ and* 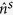, *respectively*, *and* 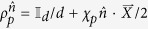 *is the probe state*, *with χ*_*p*_ *solution of the equation* 
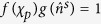
.

The proof is given in Methods. Equipped with this lemma, we are now in position to present our central result.

**Theorem 1.** *If the transformation matrix elements T*_*k*0_ = 0 *for k* ∈ {1, 2, …, *d*^2^ − *d*}, *then the evolution of* 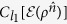 *obeys the following FR*


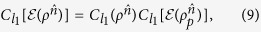


*with* 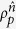 *the probe state*, *and* 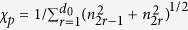.

The proof is left to the Methods. Here, we further show an implication of it. As *T*_*k*0_ = 0 for *k* ∈ {1, 2, …, *d*^2^ − *d*}, we have 
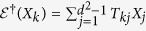
, hence 
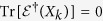
. On the other hand, 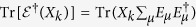. This, together with Eq. (2), requires that all the nondiagonal elements of 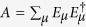 must be zero.

**Corollary 1.** *If the operator* 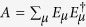 *is diagonal*, *then the evolution of* 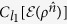 *obeys the FR* (9).

This corollary means that in addition to the usual completeness condition 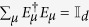 of the CPTP map^31^, the FR (9) further requires 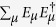 to be diagonal. We denote this kind of channels 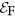. Clearly, they include the unital channel 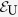 [i.e., 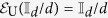] as a special case.

From a geometric perspective, Theorem 1 indicates that for all states of the same family 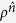, namely, states with the characteristic vectors 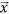 along the same or opposite directions, their coherence dynamics measured by the *l*_1_ norm can be represented qualitatively by that of the probe state 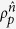, as the magnitude of 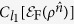 equals the product of the initial coherence 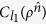 and the evolved coherence 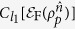. This simplifies greatly the assessment of the decoherence process of an open system. Moreover, the FR (9) provides a strong link between amount of the coherence loss of a system and structures of the applied quantum channels. Particularly, as 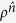 with the vectors 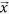 along the same or opposite directions fulfill the same decoherence law, the approach adopted here may offer a route for better understanding the interplay between geometry of the state space and various aspects of its quantum features. It might also provides a deeper insight into the effects of gate operation in quantum computing and experimental generation of coherent resources in noisy environments, as 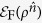 can specify the actions of environments, of measurements, or of both on the states 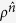.

When some restrictions are imposed on the quantum channels, the FR (9) can be further simplified.

**Corollary 2.** *If a channel* 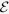 *yields* 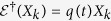 *for* 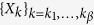 (*β* ≤ *d*^2^ − *d*), *with q*(*t*) *containing information on* 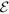
’*s structure*, *then the FR*


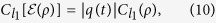


*holds for the family of states* 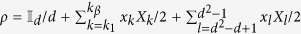.

The proof of this corollary is direct. As 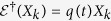, the parameters 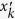 for 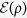 are given by 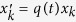. Therefore, by Eq. (3) we obtain 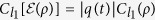. Clearly, its evolution is solely determined by the product of the initial coherence and a noise parameter |*q*(*t*)|.

There are many quantum channels satisfying the condition of Corollary 2. For instance, the Pauli channel 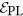 and Gell-Mann channel 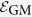 given in ref. 41, and the generalized amplitude damping channel 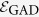
31. Notably, 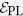 covers the bit flip, phase flip, bit-phase flip, phase damping, and depolarizing channels which embody typical noisy sources in quantum information, while 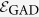 covers the structured reservoirs with Lorentzian and Ohmic-type spectral densities.

One can also construct quantum channel 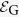 under the action of which 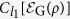 obeys the FR (10) for arbitrary initial state. The Kraus operators describing 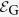 are given by


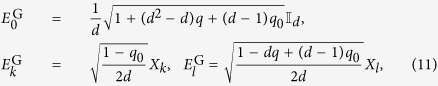


with *k* ∈ {1, …, *d*^2^ − *d*}, and *l* ∈ {*d*^2^ − *d* + 1, …, *d*^2^ − 1}, while *q* and *q*_0_ are time-dependent noisy parameters. Clearly, 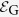 reduces to the depolarizing channel when *q*_0_ = *q*.

### *N*-qubit case

A general *N*-qubit state can be written as 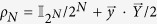, with 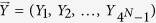, and


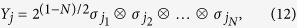


here, 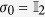, and *σ*_1,2,3_ are the usual Pauli matrices, while *j*_*k*_ takes the possible values of {0, 1, 2, 3} other than the special case *j*_*k*_ = 0 for all *k*. In the Methods section, we have proved that for every family of the *N*-qubit states 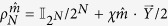, with 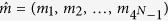 being a given unit vector, one can construct an auxiliary channel 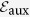 such that 
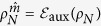
. This, together with Eq. (9), gives:

**Corollary 3.** *For any N-qubit state ρ*_*N*_, *there exists an auxiliary channel* 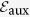 *such that*


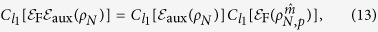


*with* 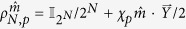, 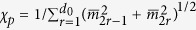, *d*_0_ = (4^*N*^ − 2^*N*^)/2, *and* 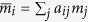, *with a*_*ij*_ *being determined by the transformation between* {*Y*_*j*_} *and* {*X*_*i*_}: 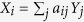.

This corollary generalizes the FR (9) for the *N*-qubit states. It shows that coherence of the evolved state under the actions of two cascaded channels 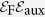 is determined by the product of the coherence for the evolved probe state under the action of 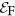 and the coherence for the generated state by 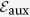. As every *Y*_*j*_ can always be decomposed as linear combinations of the generators {*X*_*i*_}, the above result applies also to the qudit states with *d* = 2^*N*^. As an explicit example, the transformation between {*Y*_*j*_} and {*X*_*i*_} for *N* = 2 is given in the Methods section, from which 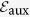 and {*a*_*ij*_} can be constructed directly.

### Frozen coherence

By Theorem 1 we can also derive conditions on the quantum channel for which the *l*_1_ norm of coherence is frozen. To elucidate this, we return to Eq. (9), from which one can see that 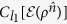 is frozen if the coherence of the probe state remains constant 1 during the evolution, i.e., 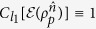. For later use, we denote by *T*^*S*^ the submatrix of *T* consisting *T*_*ij*_ with *i* ranging from 1 to *d*^2^ − *d* and *j* from 1 to *d*^2^ − 1. Then by Theorem 1 and the reasoning in its proof, we obtain the fourth corollary.

**Corollary 4.** *If T*_*k*0_ = 0 *for k* ∈ {1, 2, …, *d*^2^ − *d*}, *and T*^*S*^ *is a rectangular block diagonal matrix*, *with the main diagonal blocks*


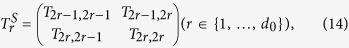


*being orthogonal matrices*, *i*.*e*., 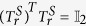, *the l*_1_ *norm of coherence for* 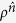 *will be frozen during the entire evolution*.

The proof is given in Methods. It enables one to construct channels 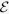 for which the *l*_1_ norm of coherence is frozen. As an explicit example, we consider the one-qubit case, with 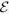 being described by 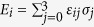, *i* ∈ {0, 1, 2, 3} and 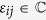. Then by Corollary 4, one can obtain that when *ε*_*i*0_ = *ε*_*i*3_ = 0, and
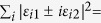

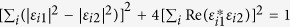
, or when *ε*_*i*1_ = *ε*_*i*2_ = 0, and 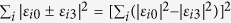 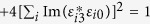, with Re(·) and Im(·) representing, respectively, the real and imaginary parts of a number, the *l*_1_ norm of coherence will be frozen. There are a host of {*ε*_*ij*_} that fulfill the requirements, e.g., *ε*_01_ = *q*(*t*), 
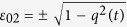
, *ε*_*k*1_ = *ε*_*k*2_ = 0, or *ε*_00_ = *q*(*t*), 
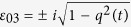
, *ε*_*k*0_ = *ε*_*k*3_ = 0, with *k* ∈ {1, 2, 3}, and *q*(*t*) contains the information on 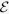
’s structure and its coupling with the system.

Moreover, for certain special initial states, the freezing condition presented in Corollary 4 may be further relaxed. In fact, for 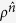 with certain *n*_2*r*−1_ = 0 (or *n*_2*r*_ = 0), 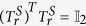 simplifies to 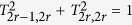 (or 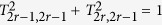). For instance, when considering the channel 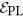
41, the *l*_1_ norm of coherence for 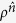 with *n*_2_ = 0 is frozen during the entire evolution when *q*_1_ = 1 (i.e., the bit flip channel). Similarly, for 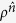 with *n*_1_ = 0, it is frozen when *q*_2_ = 1 (i.e., the bit-phase flip channel). These are in facts the results obtained in ref. 21. Needless to say, when 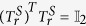, the *l*_1_ norm of coherence is also frozen for 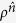 with certain *n*_2*r*−1_ = 0 or *n*_2*r*_ = 0.

### Outlook

The FR (9) presented here can be of direct relevance to other issues of quantum theory. For example, the *l*_1_ norm of coherence is a monotone of the entanglement-based coherence measure for one-qubit states^12^. Its logarithmic form 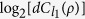 is lower bounded by the relative entropy of coherence *C*_*r*_(*ρ*) which has a clear physical interpretation, while 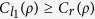 for arbitrary *ρ* has also been conjectured^46^. Further study shows that 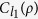 also bounds the robustness of coherence, i.e., 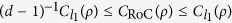
42. It is also connected to the success probability of state discrimination in interference experiments^29^ and the negativity of quantumness^21^,^47^. Thus, our results provide a route for inspecting the interrelations between decay behaviors of coherence, quantumness, and entanglement.

The FR also applies to other related coherence measures, as well as quantum correlations which are relevant to coherence. Some examples are as follows (see Methods section for their proof): (i) the coherence concurrence for one-qubit states^14^, and the trace norm coherence for one-qubit and certain qutrit states^13^,^46^; (ii) the genuine quantum coherence (GQC) defined via the Schatten *p*-norm for all states^48^, which is related to quantum thermodynamics and the resource theory of asymmetry; (iii) the robustness of coherence for the one-qubit states and *d*-dimensional states with *X*-shaped density matrix, and its lower bound 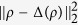 which is a measure of the GQC for all states^42^; (iv) the *K* coherence defined based on the Wigner-Yanase skew information^11^, although it is problematic in the framework of coherence by Baumgratz *et al*.^49^, it may be a proper measure of the GQC^48^; (v) the purity of a state which is complementary with quantum coherence^28^; (vi) the geometric discord^50^,^51^,^52^,^53^,^54^ and measurement-induced nonlocality^55^,^56^; (vii) the maximum Bell-inequality violation^57^, and average fidelity of remote state preparation^58^ and quantum teleportation^59^. All these manifest the universality of the FR formulated in this paper, and will certainly deepen our understanding of the already rich and appealing subject of quantum channels or the CPTP maps.

Recently, Jing *et al*. studied quantum speed limits to the rate of change of quantumness measured by the non-commutativity of the algebra of observables^60^. We note that the coherence quantifiers can also be considered as a measure of quantumness, but it is different from the notion of quantumness considered in ref. 60 and references therein, although they both characterize global quantum nature of a state, and are intimately related to quantum correlations such as discord. The coherence monotones characterize quantumness of a single state. It is basis dependent, and vanishes for the diagonal states. The quantumness based on the non-commutativity relations measures the relative quantumness of two states. It is basis independent, and vanishes only for the maximally mixed states. Of course, it is as well crucial to study evolution equation of it in future work.

## Discussion

We have established a simple FR for the evolution equation of the *l*_1_ norm of coherence, which is of practical relevance for assessing coherence loss of an open quantum system. For a general *d*-dimensional state, we determined condition such that this FR holds. The condition can be described as a restriction on the transformation matrix, or on the operator 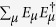, of the quantum channel. By introducing an auxiliary channel, we further presented a more general relation which applies to any *N*-qubit state. With the help of the FR, we have also determined a condition the transformation matrix should satisfy such that the *l*_1_ norm of coherence for a general state is dynamically frozen, and constructed explicitly the desired channels for one-qubit states. Finally, we showed that the FR holds for many other related coherence and quantum correlation measures. We hope these results may help in understanding the interplay between structure of the quantum channel, geometry of the state space, and decoherence of an open system, as well as their combined effects on decay behaviors of various quantum correlations.

## Methods

**Proof of Lemma 1.** As 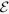 gives the map 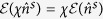, and 
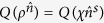
 fulfills 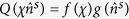, we have


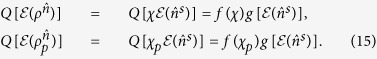


Hence, it is evident that 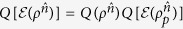 when 
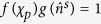
.

If *Q*_max_ ≥ 1, the equation 
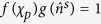
 with respect to *χ*_*p*_ is always solvable as 
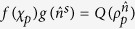
. If *Q*_max_ < 1, one can normalize it by simply introducing a constant *N* such that 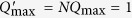, with *Q*′ obeying the FR of Eq. (8).

**Proof of Theorem 1.** First, by using Eq. (3) and the fact that 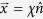, we obtain


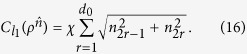


which corresponds to 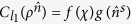, with *f*(*χ*) = *χ* and 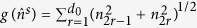.

Second, when the transformation matrix elements *T*_*k*0_ = 0 for *k* ∈ {1, 2, …, *d*^2^ − *d*}, we have


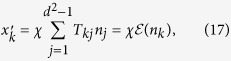


and therefore 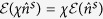.

From Eqs (16) and (17) one can see that both the *l*_1_ norm of coherence and the quantum channel 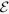 fulfill the requirements of Lemma 1, and the probe state 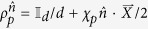, with *χ*_*p*_ being solution of the equation 
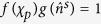
, which can be solved as 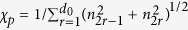. This completes the proof.

**Proof of Corollary 3.** Suppose 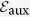 is described by the Kraus operators 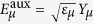, with 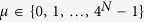. Then, by employing the anticommutation relation of the Pauli operators *σ*_1,2,3_, we obtain


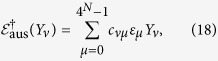


where 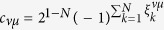, with 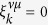 if *v*_*k*_*μ*_*k*_(*v*_*k*_ − *μ*_*k*_) = 0, and 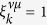 otherwise. This formula is equivalent to 
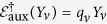
, with 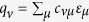 encoding the information of 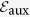.

To solve *ε*_*μ*_, we define coefficient matrix 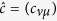, and column vectors 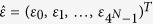, 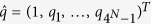, then 
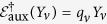
 becomes 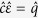, hence *ε* can be derived as 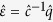, with *c*^−1^ denoting the inverse matrix of *c*. Finally, by choosing 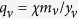, we obtain 
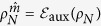
, thus completes the proof.

The transformation between generators {*Y*_*j*_} for the two-qubit states and {*X*_*i*_} for the qudit states with *d* = 4 are as follows:


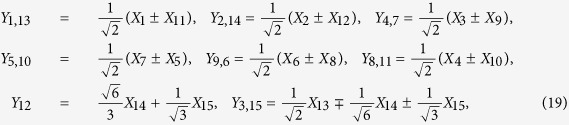


where 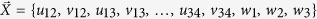, and elements 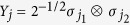 of 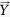 are arranged with (*j*_1_*j*_2_) in the sequence (01), (02), (03), (10), (11), (12), (13), …, (33).

**Proof of Corollary 4.** As the submatrix *T*^*S*^ is rectangular block diagonal, the elements *T*_*ij*_ in the off-diagonal blocks are all zero. This, together with *T*_*k*0_ = 0 for *k* ∈ {1, 2, …, *d*^2^ − *d*}, yields


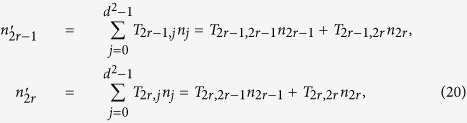


for *r* ∈ {1, 2, …, *d*_0_}. Moreover, the requirement that 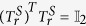 yields


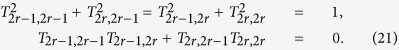


By using the above two equations, it is straightforward to see that 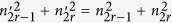, and therefore from Eq. (16) we have 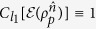. This, together with Theorem 1, implies 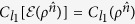, and hence completes the proof.

### Frozen coherence of one qubit

Suppose the required channel 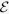 is described by the Kraus operators 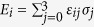, with *i* ∈ {0, 1, 2, 3}, and the values of 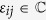 should satisfy certain constraints such that the requirement of Corollary 4 is satisfied. First, the completeness condition of the CPTP map, namely, 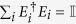
31, requires


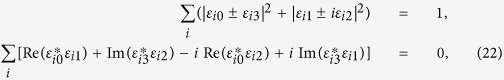


where 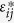 represents conjugation of *ε*_*ij*_, and the notation *i* before *ε*_*i*2_, Re(·), and Im(·) is the imaginary unit.

Second, Corollary 4 requires *T*_10_ = *T*_20_ = 0, and *T*^*S*^ to be a rectangular block diagonal matrix which corresponds to *T*_13_ = *T*_23_ = 0. This yields


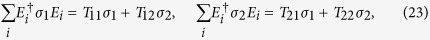


from which one can obtain
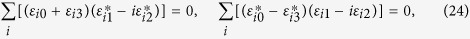
and


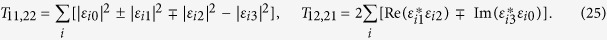


By comparing Eqs (22) and (24), one can note that the equalities are satisfied when *ε*_*i*0_ = *ε*_*i*3_ = 0, 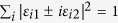, or when *ε*_*i*1_ = *ε*_*i*2_ = 0, 
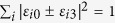
. Under these two constraints, Eq. (25) simplifies, respectively, to
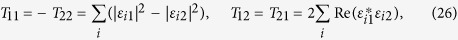
and
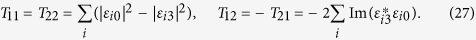


Finally, the requirement that 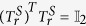, corresponds to


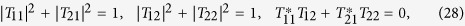


and from Eqs (26) and (27), one can see that the third equality of Eq. (28) is always satisfied, while the first two equalities are equivalent. Therefore, to freeze the *l*_1_ norm of coherence, *ε*_*ij*_ should satisfy one of the following two conditions:

(i) *ε*_*i*0_ = *ε*_*i*3_ = 0 for *i* ∈ {0, 1, 2, 3}, and


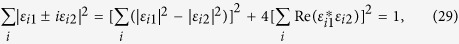


(ii) *ε*_*i*1_ = *ε*_*i*2_ = 0 for *i* ∈ {0, 1, 2, 3}, and


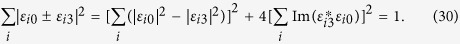


### Other measures fulfilling the FR

(i) The coherence concurrence for the one-qubit states^14^, and the trace norm coherence for the one-qubit and certain qutrit states^13^,^46^, coincide with the *l*_1_ norm of coherence. Hence, the FR applies to them.

(ii) For the GQC measure 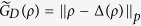 presented in ref. 48, we have


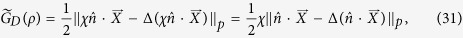


where 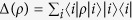 denotes full dephasing of *ρ* in the basis {|*i*〉}_*i*=1,…,*d*_. Thus, 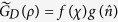, with *f*(*χ*) = *χ*/2, and 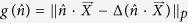.

For the GQC measure 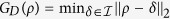, the FR also holds as the optimal *δ* is given by Δ(*ρ*)^48^.

(iii) *C*_RoC_(*ρ*) for the one-qubit states and *d*-dimensional states with *X*-shaped density matrix, equals to the *l*_1_ norm of coherence, and thus the FR holds.

(iv) The *K* coherence is defined as 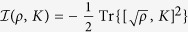
11. As 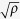 can be decomposed as 
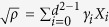
53, 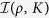 is a function of 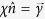, i.e., 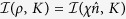, with 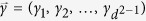. Then by using [*X*_0_, *K*] = 0, we obtain


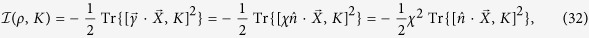


thus 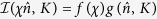, with *f*(*χ*) = −*χ*^2^/2, and 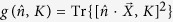.

(v) For the quantifier 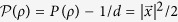 which is a monotonic function of the purity *P*(*ρ*) = Tr*ρ*^2^ of a state, we have the FR 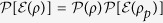, with *ρ*_*p*_ bing the probe state for which 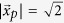.

(vi) The general form of geometric quantum correlation measure can be written as


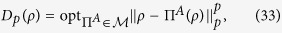


where 
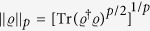
 denotes the Schatten *p*-norm, and opt represents the optimization over some class 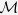 of the local measurements 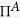. This definition covers the geometric discord^50^,^51^,^52^ and measurement-induced nonlocality^55^,^56^. For these measures, as 
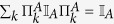
, we have


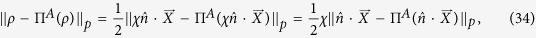


then by comparing with Lemma 1, we obtain *f*(*χ*) = (*χ*/2)^*p*^, and 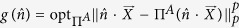, i.e., the FR holds.

If *ρ* in Eq. (33) is replaced by 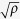, then one obtains the Hellinger distance discord for *p* = 2^53^,^54^. As 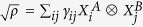, with 
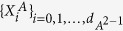
 and 
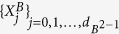
 being the sets of Hermitian operators which constitute the orthonormal operator bases for the Hilbert space 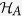 and 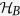
53, and 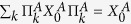, the FR also holds for it.

(vii) For two-qubit states, the maximum Bell-inequality violation *B*_max_(*ρ*)^57^, remote state preparation fidelity *F*_rsp_(*ρ*)^58^, and *N*_qt_(*ρ*) which is a monotone of the average teleportation fidelity 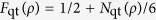
59, are given by


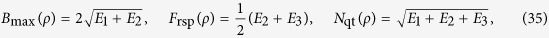


where *E*_1_ ≥ *E*_2_ ≥ *E*_3_ are eigenvalues of the 3 × 3 matrix *T*^†^*T*, and 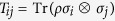. This gives 
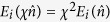
 for *i* ∈ {1, 2, 3}, which implies that all measures of Eq. (35) satisfy the requirement of Lemma 1.

## Additional Information

**How to cite this article**: Hu, M.-L. and Fan, H. Evolution equation for quantum coherence. *Sci. Rep.* **6**, 29260; doi: 10.1038/srep29260 (2016).
